# Transfusion-Transmitted Malaria: Two Pediatric Cases From the United States and Their Relevance in an Increasingly Globalized World

**DOI:** 10.1093/jpids/piab083

**Published:** 2021-09-24

**Authors:** Leigh A Stubbs, Michael Price, Daniel Noland, Jennifer Fuchs, Laura Filkins, Erin McElvania, Hung S Luu, Michael Sebert, Ami Waters, Michelle S Hsiang

**Affiliations:** 1 Department of Pediatrics, Division of Rheumatology, Baylor College of Medicine and Texas Children’s Hospital, Houston, Texas, USA; 2 Department of Pediatrics, Pediatric Residency Program, University of Texas (UT) Southwestern Medical Center, Dallas, Texas, USA; 3 Department of Pathology, UT Southwestern Medical Center, Dallas, Texas, USA; 4 Department of Pediatrics, Hospital Pediatrics Division, University of North Carolina, Chapel Hill, North Carolina, USA; 5 Department of Pathology and Laboratory Medicine, Evanston Hospital, NorthShore University HealthSystem, Evanston, Illinois, USA; 6 Department of Pediatrics, Division of Pediatric Infectious Diseases, University of Texas (UT) Southwestern Medical Center, Dallas, Texas, USA; 7 Department of Pediatrics, Division of Pediatric Hospital Medicine, University of Texas (UT) Southwestern Medical Center, Dallas, Texas, USA

**Keywords:** blood donor, *P falciparum*, *P ovale*, plasmodium

## Abstract

In non-endemic settings, transfusion-transmitted malaria (TTM) is rare but potentially fatal and becoming more common with globalization. We present two pediatric cases that demonstrate donor screening using questionnaires is subject to error and that TTM should be considered with fever following numerous transfusions in children, particularly sickle cell patients.

In non-endemic settings, malaria is mostly associated with travel or emigration from endemic areas. Less commonly, it is acquired via placental transfer, organ transplantation, or transfusions [1]. We present two cases of transfusion-transmitted malaria (TTM), which is rare but may become more common with increased globalization [2].

## CASE 1

A 14-month-old premature male was admitted with multiple respiratory viruses leading to respiratory failure requiring emergent veno-venous extracorporeal membrane oxygenation (ECMO). ECMO decannulation was complicated by right atrial perforation, hemoperitoneum, and disseminated intravascular coagulation requiring a total of 113 transfusions, including 48 aliquots of packed red blood cells (PRBCs).

**Figure 1.**
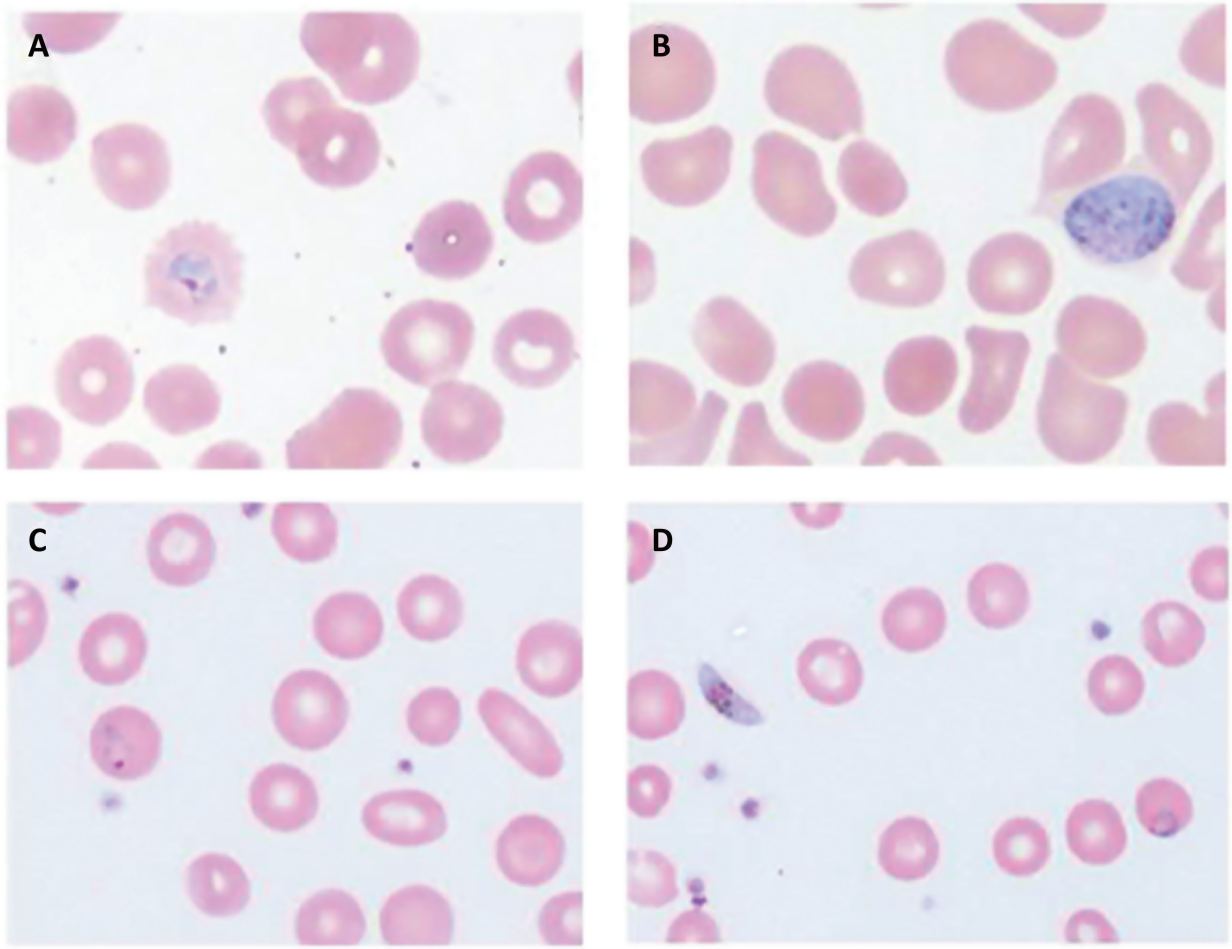
Thin smears for case 1 (A, B) and case 2 (C, D) with Wright-Giemsa staining. (A) Thin smear showing ring forms (large chromatin, sturdy cytoplasm) and red blood cells (RBC) (normal to 1.25× size, fimbriated walls) characteristic for *Plasmodium ovale*. (B) Thin smear also showing young gametocyte (round to oval) characteristic for *P. ovale*. (C) Thin smear showing ring form (delicate cytoplasm) and red blood cells (normal size) characteristic for *P. falciparum*. (D) Thin smear also showing gametocyte (crescent- or banana-shaped) and ring form at periphery of RBC, characteristic for *P. falciparum*.

Nine days after his last transfusion (hospital day 42), he developed fever, and empiric antibiotics were initiated. Bacterial and viral infectious workup was negative. His examination and imaging were significant for splenomegaly without evidence of pneumonia, intracardiac vegetations, or intra-abdominal abscess. Six days later, a 48-hour cycle to his fevers became apparent, and he developed pancytopenia and elevated inflammatory markers. A peripheral blood smear was requested to evaluate for hemophagocytic lymphohistiocytosis. *Plasmodium* parasites were incidentally identified. A thin smear revealed 0.3% parasitemia with morphology consistent with *P. vivax* or *P. ovale* (Figure 1). The patient responded well to treatment with hydroxychloroquine. Primaquine was given after glucose-6-phosphate dehydrogenase (G6PD) enzyme testing was normal. *P. ovale* was detected by polymerase chain reaction (PCR) at ARUP Laboratories and confirmed by the US Centers for Disease Control and Prevention (CDC). Further subspecies identification by CDC identified *P. ovale curtisi*. Given the lack of travel to a malaria-endemic area, a diagnosis of TTM was made.

The source investigation focused on 30 red cell donors, of which three reported travel or emigration from malaria-endemic areas. Only one had positive malaria testing. This donor mistakenly reported travel, rather than emigration from Cameroon 2 years prior, and was not appropriately deferred (deferrals were previously 1 year for any travel vs 3 years for emigration) [3]. The patient received multiple PRBC aliquots from this donor 9–18 days before onset of fever. The donor was asymptomatic before and after donation. Donor blood retained from the transfused unit tested negative by PCR (at ARUP and CDC, respectively), likely due to low-level blood-stage infection [4]. Also, there was likely DNA degradation in the retained blood sample due to storage for several weeks at 4°C before testing. However, serology testing of donor plasma by CDC was positive for *P. ovale* and *P. falciparum*, suggesting prior infection with both species, but relapsing *P. ovale* as the source [5, 6].

## CASE 2

A 13-year-old male with transfusion-dependent Hemoglobin (Hgb) SS sickle cell anemia repeatedly presented to emergency departments with fever, chills, headache, body aches, abdominal pain, emesis, and diarrhea over a week. Infectious workup detected only *Mycoplasma pneumoniae* by respiratory PCR panel, and azithromycin was prescribed. On day 7 of illness, he was admitted for acute hypoxic respiratory failure requiring noninvasive ventilation secondary to pulmonary edema without acute chest syndrome.

Laboratories showed anemia and leukocytosis without thrombocytopenia (Table 1). Abnormal red cells on the automatic differential prompted a manual review showing 5.1% infected red blood cells consistent with *P. falciparum* (Figure 1). Due to infected red blood cell count >5%, severe anemia, and worsening hypoxia, he was diagnosed with severe malaria and treated with intravenous artesunate. His respiratory status improved during a 2-day intensive care unit (ICU) stay. He was transitioned to oral artemether-lumefantrine with complete recovery. Given the lack of travel to a malaria-endemic area, a diagnosis of TTM was made.

**Table 1.** Case Comparison

Case	Clinical Features	Risk Factors	Plasmodium Species	Donor Features	Adherence to Deferral Guidelines at Time of Donation^a^	Adherence to 2020 Updated Deferral Guidelines^a^	Outcome
1	48-hour cyclical fever, splenomegaly, pancytopenia	Massive transfusion protocol, high reticulocyte count	*P. ovale curtisi* subspecies	Emigrated from Cameroon 2 years prior to donation	No	No	Full recovery
2	Irregular fever^b^, flu-like illness, headache, muscle aches, fatigue, abdominal pain, leukocytosis, anemia, no thrombocytopenia, pulmonary edema, respiratory distress	Chronic transfusions, sickle cell anemia	*P. falciparum*	Emigrated from Nigeria 4 years prior to donation, previously treated for malaria	Yes	Yes	Full recovery

^a^For residents of non-endemic countries, deferral after travel to an endemic area was decreased to 3 months from the prior policy of 1 year. For prior residents of malaria-endemic areas who have lived in a non-endemic country for less than 3 consecutive years, deferred after visiting a malaria-endemic area was increased to 3 years from the prior policy of 1 year.

^b^ *P. falciparum* is associated with a daily cyclical fevers or irregular fevers.

The blood bank reviewed the 12 PRBC units received by the patient in the last 6 months from 12 donors. One donor had relevant exposures. This donor was from Nigeria and had childhood malaria there. He emigrated 4 years prior and had not subsequently visited a malaria-endemic country. Donor blood was negative via PCR, but serology testing was positive. Further CDC genotyping analysis indicated that the recipient was infected with a single strain of *P. falciparum*, likely from West Africa based on the molecular drug resistance profile.

## Discussion

TTM is rare in non-endemic settings [7], with about one case reported every 2 years in the United States [6, 5]. Due to malaria being intra-erythrocytic, whole blood and PRBCs are implicated in most (94%) TTM cases, but any blood component containing small quantities of erythrocytes can harbor viable parasites [3]. TTM has been reported with all 5 *Plasmodium* species that infect humans. In the United States and Europe, a predominance of *P. falciparum* is seen due to most infective donors originating from sub-Saharan Africa [2, 5]. High burdens of *P. vivax* in the Americas and Asia make this species a significant etiology of TTM in these areas [2, 8]. TTM due to *P. ovale* is rare, with only four cases previously reported [7]. With molecular testing for *P. ovale* subspecies identification recently made available, our case represents the first reported case of TTM due to *P. ovale curtisi*.

Our patients had anemia and characteristic fever patterns (Table 1). Thrombocytopenia is common with malaria and occurred in the first case, but is it uncommon with underlying HbSS, as with the second case [9]. Due to low parasite inoculums from asymptomatic donors, longer incubation periods are observed with TTM as compared to mosquito-transmitted malaria (MTM); the incubation periods for our cases were within previously reported ranges [7]. Due to direct inoculation of erythrocytic stages, parasite development bypasses the liver stage. Thus, the use of primaquine to treat hypnozoites responsible for *P. ovale* relapse was not necessary in case 1 [7].

Both patients had favorable outcomes, but the second case necessitated ICU admission. The high mortality rate (~11% in the United States) associated with TTM is likely due to delays in diagnosis and treatment as well as comorbidities and compromised host defense among transfusion recipients [5, 7]. Sickle cell patients are at higher risk of TTM due to chronic transfusions and at higher risk of death due to exacerbation of already existing anemia and impaired splenic function [10]. Age-dependent clinical immunity has been documented in endemic settings [11] and could imply that children are more likely to be symptomatic. In non-endemic settings worldwide, children account for approximately 30% of TTM cases [7]. Notably, all 4 TTM cases reported in the United States since 2011 (inclusive of our cases) occurred in children or adolescents. The other two cases were in adolescents (16 and 18 years) with sickle cell disease [1, 6].

In non-endemic settings, blood donor screening policies balance the risks of TTM against the costs of excluding healthy donors [2, 5]. In Europe and Australia, where a larger proportion of donors are at high risk (eg, 6% in France and 13.5% in Australia), selective screening is based on initial questioning, and followed by malaria testing using serological and/or molecular methods in high risk donors [2]. In the United States and Canada, where 1.4% and 3.1% of donors are at-risk, respectively, screening relies solely on reported travel and immigration history [2, 3].

Between 2000 and 2017, all reported TTM cases in the United States (n = 11) were from asymptomatically infected donors that emigrated from malaria-endemic countries [3, 6]. Considering this and due to blood supply shortages during the coronavirus pandemic, the United States revised deferral protocols in April 2020, with a longer deferral (3 years) for travel among prior residents of malaria-endemic areas who have lived in a non-endemic country for less than 3 consecutive years, and a shorter deferral for travel among residents from non-endemic countries (3 months) [3]. Using these criteria, the incorrect report of travel in the 2 years prior in case 1 would have still necessitated deferral but case 2 would not be prevented since the donor emigrated 4 years prior. Nonetheless, case 1 underscores the challenge of recall bias or donor comprehension of screening questions; in the United States, 71% of TTM cases have been reported to occur due to imperfect application of deferral guidelines [6]. The remaining 29% of the cases occurred despite guideline adherence as in our second case. Even in countries where selective screening is conducted, TTM can still occur due to reliance on questioning to trigger testing.

As TTM is rare, our pediatric institution having two cases in 3 years is notable. We hypothesize a few reasons. First, TTM may be underrecognized due to limited experience by practitioners and laboratory technologists. Incidental detection in our cases can be attributed to our hospital having highly skilled laboratory technologists. Second, children versus adults may be more likely to be symptomatic, which prompts additional workup leading to diagnosis. Third, among US cities, the Dallas-Fort Worth-Arlington Metroplex has among the highest concentration of immigrants from sub-Saharan Africa, thus increasing the likelihood of asymptomatically infected donors [12]. We additionally considered whether local screening processes pose a greater risk than the national average, but our second case would not have been prevented by even perfect adherence to current deferral criteria.

Our cases illustrate that blood donor screening using questionnaires is subject to error, despite recent changes in deferral criteria. In an increasingly globalized world, TTM should be considered with fever following numerous transfusions in children, particularly sickle cell patients. Awareness by pediatricians, infectious diseases specialists, and laboratory technologists can facilitate early diagnosis and treatment to prevent potentially fatal outcomes.

## Notes

***Acknowledgments**.* Thank you to Naomi Lucchi, Dragan Ljolje, Hilda Rivera, Sukwan Handali, and other staff from the US CDC for their assistance in the malaria molecular and serological testing and review of the paper. We also thank Drs. Amanda Evans and Patricia Pichilingue Reto for their clinical inputs to the case.

***Financial support**.* This work was supported by the Horchow Family Fund Scholarship in the Department of Pediatrics at UT Southwestern Medical Center (Grant number 5300375400 to M.S.H.).

***Financial disclosure**.* The authors have no other financial relationships relevant to this article to disclose.

***Potential conflicts of interest**.* All authors: No reported conflicts.
